# Dual cardiac amyloidosis with AL cardiomyopathy and ATTR cardiomyopathy in systemic AL amyloidosis: Recommendation for a myocardial histology-based diagnosis

**DOI:** 10.1016/j.jccase.2026.06.013

**Published:** 2026-07-09

**Authors:** Kazuki Fujioka, Tetsuji Kitano, Naoki Fujimoto, Kazuaki Maruyama, Kai Miyazaki, Yukako Shintani-Domoto, Kyoko Imanaka-Yoshida, Kaoru Dohi

**Affiliations:** a Department of Cardiology and Nephrology, Mie University Graduate School of Medicine, Tsu, Japan; b Department of Cardiology, Matsusaka Chuo General Hospital, Matsusaka, Japan; c Department of Pathology and Matrix Biology, Graduate School of Medicine, Mie University, Tsu, Japan; d Department of Integrated Diagnostic Pathology, Nippon Medical School, Tokyo, Japan

**Keywords:** Amyloid, Dual amyloidosis, Cardiomyopathy, Transthyretin amyloid cardiomyopathy, λ-Type light chain cardiomyopathy

## Abstract

Accurate amyloid typing in cardiac amyloidosis is clinically important for determining treatment strategies. A 73-year-old man underwent a renal biopsy due to renal dysfunction and was diagnosed with systemic λ-type light chain (AL) amyloidosis. Echocardiography and cardiac magnetic resonance revealed cardiac involvement, leading to a clinical diagnosis of λ-type AL-cardiomyopathy (CM). However, the clinical course and findings were inconsistent with AL-CM. Technetium-99m-pyrophosphate scintigraphy showed intense myocardial uptake corresponding to Grade 3 on visual assessment. Further examination through an endomyocardial biopsy and protein mass spectrometry revealed the patient had both AL-CM and transthyretin amyloid (ATTR)-CM. In addition to chemotherapy for systemic AL amyloidosis, transthyretin stabilizing therapy was initiated for wild-type ATTR-CM. Initially, this patient was diagnosed with AL-CM complicated by systemic AL amyloidosis; however, it was subsequently determined that the cardiac involvement was a coexistence of both AL-CM and ATTR-CM.

**Learning objective:**

Even though multimodality imaging approaches have been established with recent advances in imaging technology, it is still challenging to noninvasively and accurately determine specific amyloid subtypes or to predict the presence of multiple types of amyloids. Accurate amyloidosis typing is critically important for determining treatment strategies. When clinical course and imaging findings conflict with the diagnosis, diagnosis based on myocardial tissue should not be deferred. Furthermore, proteomic analysis is highly useful for accurate amyloid typing.

## Introduction

Cardiac amyloidosis is a progressive disease that causes cardiac dysfunction due to the deposition of amyloid fibers in the myocardium. Although nine types of amyloidogenic proteins accumulate in the myocardium to cause significant cardiac disease, over 98% of cardiac amyloidosis diagnoses result from fibrils composed of light chain (AL) or transthyretin (ATTR) [1]. The gold standard for diagnosing cardiac amyloidosis is an endomyocardial biopsy. The European Society of Cardiology Myocardial Working Group has proposed an alternative diagnosis based on a combination of amyloid deposition in an extracardiac biopsy and typical imaging findings from echocardiography or cardiac magnetic resonance (CMR) [1]. However, the guidelines assume that a patient has a single type of amyloidosis and may not consider the possibility of multiple types. We have experienced a case in which accurate amyloid typing of cardiac involvement in a patient diagnosed with systemic AL amyloidosis was achieved not only through noninvasive imaging but also by performing tissue biopsy and proteomics analysis.

## Case report

A 73-year-old man was referred to the nephrology department two months previously with a history of progressive renal dysfunction, hypoalbuminemia, and bilateral lower extremity edema for 1.5 years. He was diagnosed with nephrotic syndrome, with the presence of serum M-protein and urinary Bence Jones protein, suggesting systemic amyloidosis. Bone marrow examination showed no clonal proliferation of plasma cells, ruling out multiple myeloma. Both the renal and gastric biopsies revealed Congo red-positive amyloid deposits. Immunohistochemical staining of renal tissue showed positive deposition of λ light chain in area corresponding to the amyloid deposits but was negative for TTR and kappa (ƙ) light chain (Fig. 1A–D). This led to a diagnosis of systemic AL. Chemotherapy with the daratumumab, cyclophosphamide, bortezomib, dexamethasone regimen was initiated.

**Fig. 1:**
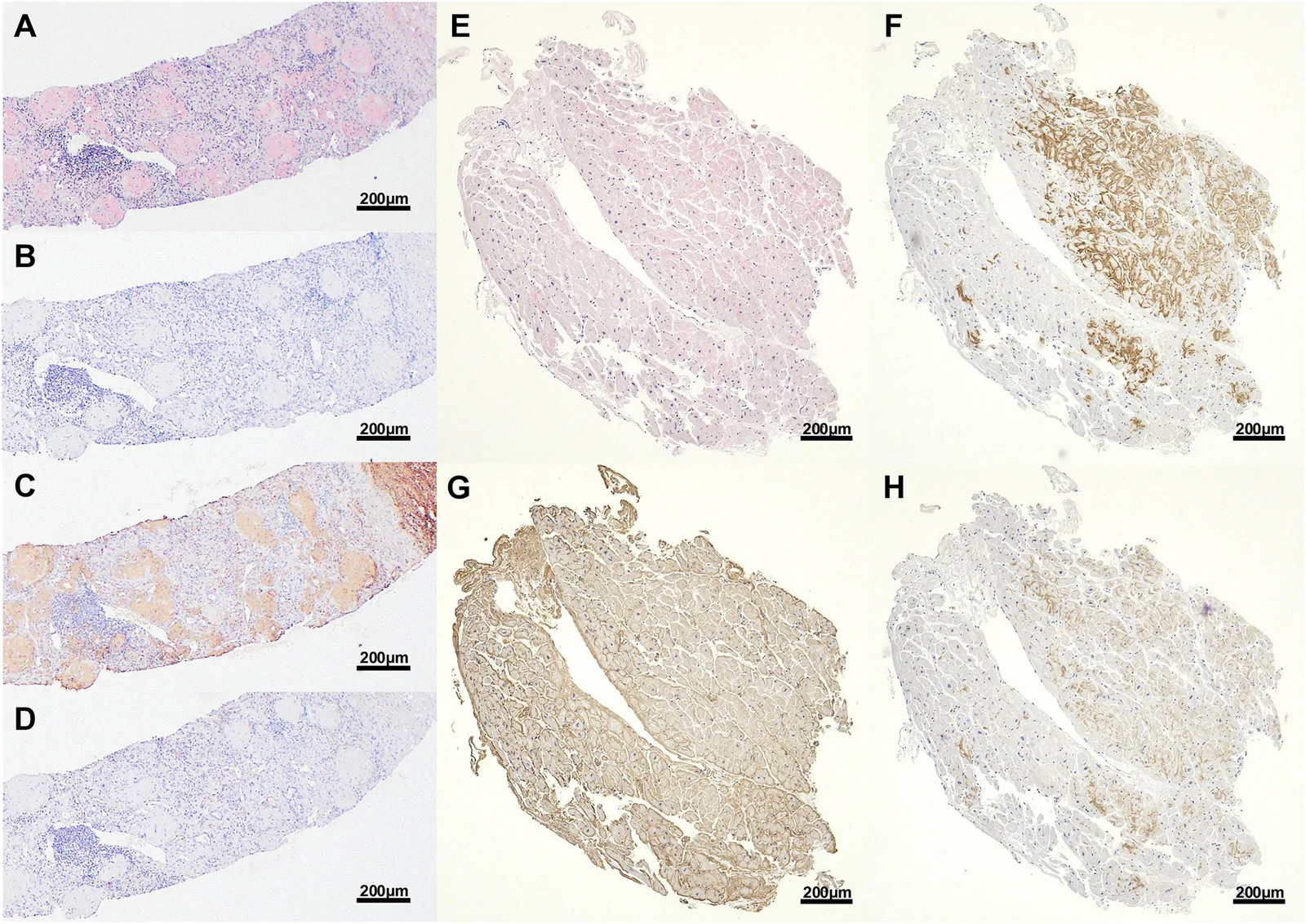
Renal and myocardial biopsy. (A–D) Renal tissue. (E–H) Myocardial tissue. Congo red (A, E), transthyretin (TTR) (B, F), amyloid lambda (λ) light chain (C, G), and amyloid kappa (ƙ) light chain (D, H). In renal tissue, only λ-type light chains showed deposition in the region corresponding to Congo red staining. In contrast, both TTR and λ-type light chains showed accumulation in the area corresponding to Congo red staining in myocardial tissue.

**Fig. 2:**
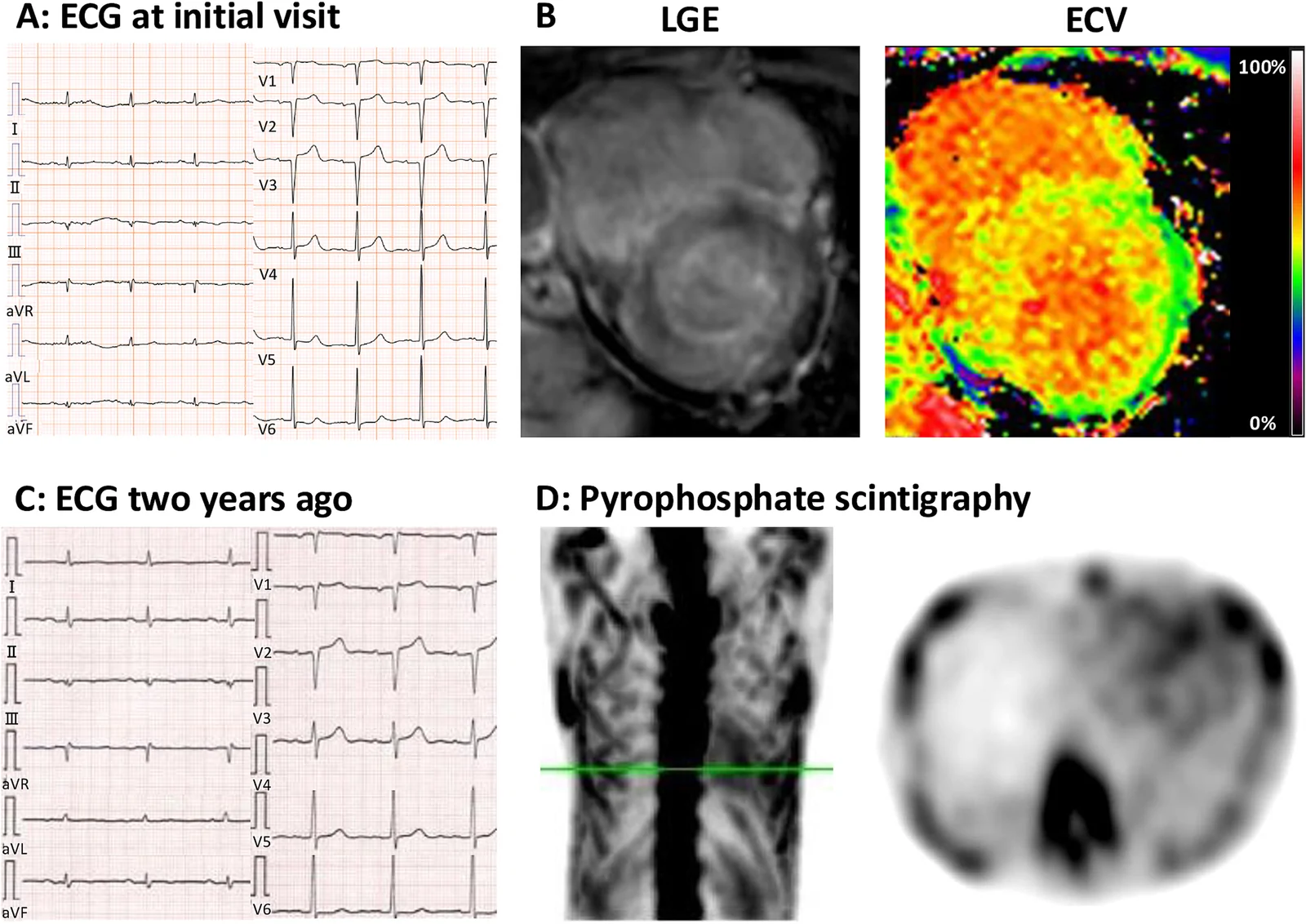
Examination findings. The initial electrocardiography (ECG) (A) showed first-degree atrioventricular block, low voltage in the limb leads, and poor R-wave progression in the chest leads. Late gadolinium enhancement (LGE) on cardiac magnetic resonance imaging (B) showed extensive involvement from subendocardial to mid-myocardial layers of the left ventricular myocardium. The extracellular volume (ECV) was 49.4%. The ECG with local clinic two years previously (C) showed low voltage in the limb leads, PR interval prolongation, and poor R-wave progression in the chest leads. Coronal and transverse view (D) of Technetium-99m-pyrophosphate scintigraphy showed intense myocardial uptake corresponding to Grade 3 on visual assessment, with a heart-to-contralateral lung ratio at 3 h post-injection of 1.38.

**Fig. 3:**
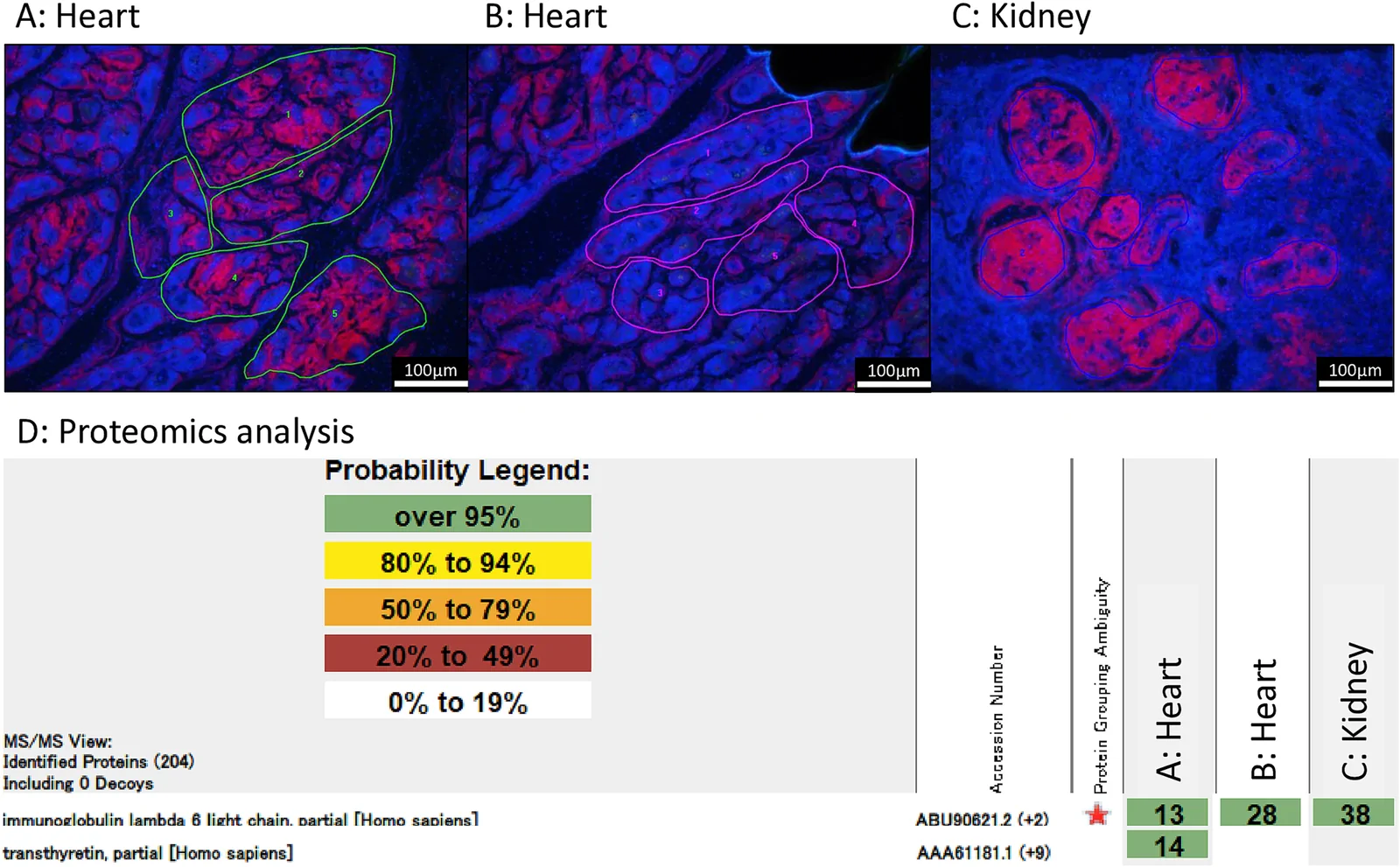
Proteomic analysis. The top panel, (A–C), shows representative Congo red-stained images viewed under a fluorescence filter of the three regions subjected to laser microdissection followed by liquid chromatography mass spectrometry (LC-MS). Each region was selected based on the immunohistochemistry (IHC) positivity in the corresponding serial section. The serial section corresponding to (A) shows thick TTR-IHC-positive amyloid deposits in the myocardial interstitium, whereas (B) shows thin λ-IHC-positive interstitial myocardial deposits, and (C) shows λ-IHC-positive renal deposits. The results of the proteomic analysis are shown as total spectrum count in (D) (Scaffold, Proteome Software). The top entry in (D) “immunoglobulin lambda 6 light chain, partial [*Homo sapiens*],” represents the λ-type light chain, which was detected in all three regions (A–C). The bottom entry, “transthyretin, partial [*Homo sapiens*],” represents TTR amyloid, detected exclusively in the region shown in (A).

He was then referred to our cardiology department for further evaluation of potential cardiac involvement. He exhibited bilateral lower leg edema but had no subjective heart failure symptoms. His medical history included diabetes mellitus and complete blindness of unknown etiology, but he had no history of carpal tunnel syndrome or spinal stenosis. His blood pressure was 124/74 mmHg, and pulse rate was 68 beats/min. Heart sounds were regular, with a fourth heart sound audible. No macroglossia was noted. Phalen's and reverse Phalen's signs were negative. B-type natriuretic peptide level was elevated to 122.2 pg/mL (Online Table 1). Electrocardiography (ECG) demonstrated sinus rhythm with first-degree atrioventricular block, low voltage in limb leads, and poor R-wave progression in leads V1–V3 (Fig. 2A). Transthoracic echocardiography revealed preserved left ventricular (LV) wall motion, diffuse LV hypertrophy, and Grade 1 diastolic dysfunction. However, no apical sparing pattern was observed. CMR revealed no impairment in LV systolic function, however, concentric LV myocardial hypertrophy with diffuse subendocardial to midmyocardial late gadolinium enhancement. Native T1 mapping showed a markedly elevated native T1 value [1468 ms (institutional reference range: 1280 ± 36 ms)] and extracellular volume (49.4%) (Fig. 2B). These findings were consistent with cardiac amyloidosis. Therefore, since light chain deposits had already been identified in several other organs, it was considered that AL-cardiomyopathy (CM) was also present.

During several months of follow-up, subjective symptoms, physical examinations, and echocardiographic findings remained unchanged from the time of diagnosis (Online Table 1). Although CMR findings suggested extensive myocardial fibrosis and severe amyloid deposition, the patient did not require diuretics and did not develop decompensated heart failure. This course of the disease was inconsistent with the typical clinical course of AL-CM, which is generally characterized by rapid progression and a poor prognosis. In addition, an ECG taken at a local clinic two years prior to the onset of renal dysfunction (Fig. 2C), already showed findings similar to those observed at the initial visit, including first-degree atrioventricular block, low voltage, and poor R-wave progression. Based on these findings, we suspected that the cardiac involvement might be due to ATTR-CM rather than AL-CM, or that both conditions might coexist. Technetium-99 m-pyrophosphate (Tc-99m-PYP) scintigraphy showed intense myocardial uptake corresponding to Grade 3 on visual assessment (Fig. 2D). Given the possibility of coexisting ATTR-CM, an endomyocardial biopsy was performed from the right ventricular septum. Congo red staining revealed amyloid deposits (Fig. 1E). Immunohistochemical staining showed TTR and λ-type light chain positively at the sites of amyloid deposition (Fig. 1F, G), while ƙ-type light chain staining was negative (Fig. 1H). Based on the immunohistochemistry (IHC) results, dual amyloidosis was suspected. To achieve accurate amyloid typing and to clarify the cause of the systemic symptoms, proteomic analysis using laser microdissection was conducted [2]. We analyzed two regions from the myocardial tissue— thick TTR-IHC-positive myocardial interstitium (Fig. 3A) and thin λ-type light chain-IHC-positive myocardial interstitium (Fig. 3B) — and one region from the λ-type light chain-IHC-positive renal tissue (Fig. 3C). TTR was detected exclusively in the TTR-IHC-positive region, whereas a concordant clonotype λ-type light chain was detected in all three regions, including the TTR-IHC-positive region. Genetic testing revealed no mutations in the TTR gene. Based on these results, the patient was diagnosed with systemic AL amyloidosis complicated by both λ-type AL-CM and wild-type ATTR-CM. Chemotherapy for systemic AL amyloidosis was continued, and TTR stabilization therapy was initiated for wild-type ATTR-CM.

## Discussion

To our knowledge, this is a rare case of dual cardiac amyloidosis in a patient with systemic AL amyloidosis presenting with nephrotic syndrome.

A previous study reported that of 1094 patients who had been diagnosed with amyloidosis, 9 patients presented with two different amyloid types in at least two separate specimens [3]. Although the occurrence of two different types of amyloidosis in individual patients is an uncommon phenomenon, similar cases have been increasingly reported in recent years, probably because of the world-wide rise in the use of amyloid-modifying medications. Given the significant differences in therapeutic agents and prognosis between ATTR-CM and AL-CM, as well as the recent advances in disease-modifying therapies for ATTR-CM and the availability of antibody preparations for AL-CM, it is crucial to consider the possibility of coexistence of the multiple types of amyloidosis.

In the present case, the patient was diagnosed with systemic AL amyloidosis following renal dysfunction, and the cardiac involvement was initially thought to be AL-CM based on non-invasive imaging. Notably, despite CMR findings showing extensive fibrosis and amyloid deposition, no clinical deterioration was observed during follow-up after the diagnosis of AL-CM. Furthermore, an ECG recorded two years prior to the diagnosis of AL-CM already revealed low voltage and conduction abnormalities similar to those observed at the time of diagnosis. This clinical course appeared atypical for AL-CM, which is characterized by rapid progression of cardiac involvement and a poor prognosis [4]. Consequently, Tc-99 m-PYP scintigraphy and endomyocardial biopsy were performed, and based on immunohistochemical staining and proteomic analysis of endomyocardial tissue, the patient was finally diagnosed with dual cardiac amyloidosis. It is impossible to determine which type of cardiac amyloidosis is predominant even using myocardial tissue or proteomic analysis.

Proteomic analysis using laser microdissection followed by liquid chromatography–tandem mass spectrometry (LC-MS/MS) was performed using a workflow established in our laboratory [2], based on the methods reported by Vrana et al. [5] and Sethi et al. [6], originally developed by Dogan and colleagues at the Mayo Clinic. The detailed protocol established in our laboratory is described in the Online Methods.

Recent advances in diagnostic imaging technology have led to reports indicating that T2 values on CMR are higher in AL-CM (56.6–63.2 ms) than in ATTR-CM (54.2–56.2 ms). Although multimodality imaging approaches have been established [7], it is still challenging to noninvasively and accurately determine specific amyloid subtypes or to predict the presence of multiple types of amyloids. In particular, when diagnosing cardiac amyloidosis based on extracardiac biopsy positive for amyloid and typical echocardiography and CMR findings without performing myocardial biopsy, there is a risk of misdiagnosing the amyloid type. If the patient's medical history or clinical presentation appear atypical for the initial diagnosis or are not fully explained by the initial diagnosis, we should not defer myocardial tissue-based diagnosis, considering the possibility of dual amyloidosis or the presence of different amyloid types in extracardiac and myocardial tissues. Precise amyloidosis typing is very important for decision-making in the treatment strategy.

## Consent

Written informed consent was obtained from the patient.

## Declaration of competing interest

The authors declares that there is no conflict of interest.

## Appendix A Supplementary data

Online Table 1 Summary of patient characteristics
