# Supplementary material for: Dual cardiac amyloidosis with AL cardiomyopathy and ATTR cardiomyopathy in systemic AL amyloidosis: Recommendation for a myocardial histology-based diagnosis
Source: J Cardiol Cases. 2026 Jul 9;34(4):157–60. doi: 10.1016/j.jccase.2026.06.013 (PMC13670378; doi:10.1016/j.jccase.2026.06.013)
Supplement: Supplementary file 1 [file mmc1.docx]

**Table 1** Summary of patient characteristics.

| Variables | At initial visit | Six months later  (During hospitalization for cardiac biopsy) |
| --- | --- | --- |
| Height (cm) | 157 | 157 |
| Weight (kg) | 44.6 | 46.4 |
| Body mass index (kg/m^2^) | 18.1 | 18.8 |
| Systolic blood pressure (mmHg) | 116 | 124 |
| Diastolic blood pressure (mmHg) | 73 | 74 |
| Heart rate (beat per minute) | 76 | 68 |
| **Laboratory tests** |  |  |
| White blood cell count (/µL) | 12.2*10^3^ | 4.1*10^3^ |
| Hemoglobin (g/dL) | 13.7 | 9.4 |
| Platelet count (/µL) | 292*10^3^ | 136*10^3^ |
| Total protein (g/dL) | 5.0 | 4.2 |
| Albumin (g/dL) | 1.5 | 2.3 |
| Creatinine (mg/dL) | 1.2 | 1.3 |
| Creatinine clearance (mL/min) | 34.5 | 34.2 |
| Sodium (mEq/L) | 139 | 140 |
| Potassium (mEq/L) | 4.0 | 3.8 |
| Calcium (mEq/L) | 7.8 | 8.0 |
| BNP (pg/mL) | 122.2 | 164.3 |
| High-sensitivity troponin I (ng/mL) | 143 | NA |
| High-sensitivity troponin T (ng/mL) | NA | 0.078 |
| Urine protein-to-creatinine ratio (g/gCr) | 10.8 | 9.0 |
| λ light chain(mg/L) | 176.0 | 8.6 |
| ƙ light chain(mg/L) | 53.6 | 1.4 |
| ƙ / λ ratio | 0.30 | 0.16 |
| SIE | IgG-λ type M protein | IgG-ƙ type M protein |
| UIE | Bence Jones protein | Negative |
| **Echocardiographic findings** |  |  |
| LVDd (mm) | 41 | 42 |
| LVDs (mm) | 26 | 28 |
| IVSth (mm) | 12 | 13 |
| PWth (mm) | 11 | 12 |
| LVEDV (mL) | 86 | 76 |
| LVESV (mL) | 33 | 29 |
| LVEF (%) | 62 | 61 |
| E wave velocity (cm/s) | 55 | 60 |
| A wave velocity (cm/s) | 89 | 89 |
| Deceleration time (ms) | 267 | 238 |
| E/A | 0.6 | 0.7 |
| e’ (cm/s) | 3.7 | 5.4 |
| E/e’ | 13.0 | 11.2 |
| Relative apical sparing ratio | 0.91 | 0.90 |

BNP, B-type natriuretic peptide; IgG, immunoglobulin G; IVSth, intraventricular septum thickness; LVDd, left ventricular end-diastolic dimension; LVDs, left ventricular end-systole dimension; LVEDV, left ventricular end-diastolic volume; LVEF, left ventricular ejection fraction; LVESV, left ventricular end-systolic volume; NA, not available; PWth, posterior wall thickness; RELAPS, relative apical sparing; SIE, serum immunoelectrophoresis; UIE, urine immunoelectrophoresis. Relative apical sparing ratio was calculated as (average apical longitudinal strain) / [(average basal longitudinal strain) + (average midventricular longitudinal strain)].
