# Supplementary material for: Dual cardiac amyloidosis with AL cardiomyopathy and ATTR cardiomyopathy in systemic AL amyloidosis: Recommendation for a myocardial histology-based diagnosis
Source: J Cardiol Cases. 2026 Jul 9;34(4):157–60. doi: 10.1016/j.jccase.2026.06.013 (PMC13670378; doi:10.1016/j.jccase.2026.06.013)
Supplement: Supplementary file 2 [file mmc2.docx]

Online Methods

According to previous reports, formalin-fixed paraffin-embedded specimens (10-µm-thick) were prepared and laser microdissection (LMD)-liquid chromatography–tandem mass spectrometry (LC-MS/MS) was performed [2]. LMD was performed to isolate Congo Red–positive regions [approximately 200,000 µm2], each area for proteomic analysis using LMD7000 (Leica Microsystems GmbH, Wetzlar, Germany). The specimens were lysed in buffer with 10 mM Tris-base/1mM EDTA/0.002% Zwittergent 3-16. Following sonication and boiling, it was digested overnight using 0.5 µg Trypsin Gold (V528A, Promega, Madison, WI, USA) at 37 °C. After adding dithiothreitol and boiling, samples were evaporated in a vacuum concentrator centrifuge system at 37°C. The residues were resuspended in water containing 0.1% formic acid and then filtered using an Ultrafree-MC unit (UFC30LG00, Merck KGaA, Darmstadt, Germany). In total, a part of the digested protein was injected into a nanoEase M/Z Symmetry C18 trap column (180 mm x 20 mm, 5 μm, 100 Å, Cat.No.186008821, Waters, Milford, MA, USA) and further separated through an ACQUITY UPLC M-Class system (Waters) attached to an injector valve for desalinating and concentrating peptides. After washing the trap column, the peptides were loaded into a nanoEase M/Z HSS C18 T3 column (75 μm x 100 mm, 1.8 μm, 100 Å, Cat.No.186008815, Waters) by switching the valve. The temperature of the column oven was set at 35°C. The mobile phase consisted of solution A (0.1% formic acid in water) and solvent B (0.1% formic acid in acetonitrile). The samples were loaded with 1% solvent B at a flow rate of 5 µl/min for 6 min and eluted from the analytical column with concentration gradient of acetonitrile from 2%B to 30%B over 90 min at a flow rate of 300 nL/min. Eluted peptides were analyzed using a ZenoTOF 7600 mass spectrometer (SCIEX, Framingham, MA, USA). Gradient-eluted peptides were analyzed using Zeno Information Dependent Acquisition (IDA), generally known as data-dependent acquisition (DDA). TOF MS survey scans were performed over 400–1250 m/z with an accumulation time of 250 msec. Subsequently, up to 40 precursor ions with charge states of 2–5 and intensities greater than 100 counts were selected for fragmentation using collision-induced dissociation. MS/MS spectra were acquired across 100–1500 m/z (50 msec accumulation time) using rolling (dynamic) collision energy. All MS/MS spectral data were analyzed using MASCOT v2.8.2 (Matrix Science, London, UK) against Swiss-Prot (2023_02). LC–MS/MS spectra were searched against the human protein database with a peptide mass tolerance of ±10 ppm and a fragment MS/MS tolerance of 0.01 Da, allowing up to two missed cleavages. Variable modifications included methionine oxidation (N-terminus), Gln→pyro-Glu (N-terminal Q), and methylation (K). The search results were filtered at a false discovery rate of 1% using the decoy search option. The resulting peptide and protein identifications were further validated and quantified using Scaffold (version 5.3.3, Proteome Software, Portland, OR, USA), which provided probability scoring and spectral count–based evaluation. The abundance of the identified proteins was expressed as total spectrum count.
